# The best execution of the DuoStim strategy (double stimulation in the follicular and luteal phase of the same ovarian cycle) in patients who are poor ovarian responders

**DOI:** 10.1186/s12958-020-00655-3

**Published:** 2020-10-15

**Authors:** Yanqun Luo, Li Sun, Mei Dong, Xiqian Zhang, Li Huang, Xiulan Zhu, Yingqi Nong, Fenghua Liu

**Affiliations:** 1 The First Affiliated Hospital of Jinan University, 613 Huangpu Avenue West, Tianhe District, Guangzhou, 510630 Guangdong Province China; 2 Department of Reproductive Medical Center, Guangdong Women and Children Hospital, No. 521 Xingnan Road, Guangzhou, 511400 Guangdong Province China

**Keywords:** Ovulation induction, Luteal phase, Follicular phase, Cryopreservation, Gonadotropin-releasing hormone

## Abstract

**Background:**

Patients found to be poor ovarian responders (POR) are a challenging patient population for any assisted reproduction technology. Despite attempts at various controlled ovarian stimulation schemes, reproductive outcomes in this patient population have not improved. In recent years, the DuoStim protocol (both follicular and luteal phase stimulation during the same menstrual cycle) has shown a potential for use in patients with POR.

**Methods:**

This retrospective study reviewed the medical records of 304 women who were diagnosed as POR and underwent the DuoStim protocol. We compared follicular phase stimulation (FPS) data and luteal phase stimulation (LPS) data of the same patients. We also compared the effects of different trigger drugs including urine human chorionic gonadotropin (uHCG; 10,000 IU), recombinant human chorionic gonadotropin (rHCG; 250 μg), and gonadotropin-releasing hormone agonist (GnRH-a; 0.2 mg) at the FPS and LPS stages.

**Results:**

POR undergoing the DuoStim protocol resulted in a significantly higher number of oocytes retrieved, normal fertilised oocytes, cleaved embryos, cryopreserved embryos, and good quality embryos at the LPS stage than at the FPS stage. Trigger drugs at the FPS stage did not affect the FPS stage data. Regardless of the stage, rHCG and GnRH-a yielded significantly more cryopreserved embryos and good quality embryos than uHCG.

**Conclusion:**

The use of GnRH-a or rHCG as the trigger drug may be better than uHCG in both the FPS and LPS stages for POR undergoing the DuoStim protocol. This will increase the number of good quality embryos at the LPS stage. We found that the LPS stage results in more oocytes (and therefore more embryos) than the FPS stage.

## Background

Poor ovarian responders (POR) are a challenging patient population in the field of assisted reproduction technology (ART). Despite attempts at various controlled ovarian stimulation (COS) protocols, a greater number of embryos or a higher quality of embryos have not been obtained in this patient population [1]. However, luteal phase stimulation and the development of whole embryo freezing technology have been proven to be safe due to their use in the fertility preservation of patients with cancer [1].

Based on the breakthrough of the follicular wave theory, [2–4] COS protocols have been updated with luteal phase simulation protocols and, more recently, the DuoStim protocol (follicular and luteal phase stimulation during the same menstrual cycle). Random start protocols have also been used. At present, the DuoStim protocol is typically used to provide fertility preservation for patients with cancer. Its applications in POR are recent developments [2]. The rapid development of cryopreservation technology and the implementation of the whole embryo freezing strategy have allowed for the DuoStim protocol to eliminate the traditional COS-oocyte retrieval-embryo transfer procedure and achieve more favourable results [5, 6].

There is currently limited research on DuoStim for POR [7–16]. This may be due to the fact that the traditional COS protocol is preferred in more clinical practices, as DuoStim is time-consuming and costly. The cancellation rate of the DuoStim procedure is very high [17, 18].

Among the published reports of DuoStim, the inclusion criteria for POR vary, as some studies adopted the Bologna criteria, [19] while others used the researchers’ custom criteria for patient inclusion. There are some previous studies that compare the DuoStim protocol with the traditional COS protocol [7–9]. Although DuoStim does not show advantages in all evaluation indicators, most of these studies tend to have better reproductive outcomes for DuoStim. The number of eggs, metaphase II (MII) oocytes, and embryos in the cleavage stage and the rate of superior embryos is increased after the DuoStim protocol compared to the traditional COS protocol. Studies that compare the embryos obtained after the follicular phase stimulation (FPS) stage with those obtained after the luteal phase stimulation (LPS) stage in DuoStim have found that stimulation during the LPS stage results in a higher number of oocytes and embryos [10–16]. However, the standards for embryo evaluation data vary widely, and the conclusions of these studies are contradictory. For the preimplantation genetic testing for aneuploidy (PGT-A) population, the DuoStim protocol has a higher euploid blastocyst rate [9] than the traditional COS protocol, and the euploid blastocyst rate obtained at the FPS stage is higher than that obtained at the LPS stage [20]. These studies focus more on the comparison of the dosage of gonadotropin (Gn), days of Gn duration, and COS protocol. While studies have shown that different trigger medicines impact embryo quality, [21] the trigger medicines of the DuoStim protocol have not been compared.

As the DuoStim protocol includes whole embryo vitrification, it is particularly important to discuss the rate of good quality embryos obtained using this method [9]. Therefore, under the premise of Bologna’s inclusion criteria, this retrospective study compares the embryos obtained at the LPS stage with those obtained at the FPS stage through comprehensive embryo assessment data, and explores the impact of different trigger medications of the DuoStim protocol on embryo data for POR.

## Methods

### Study design and participants

#### Patients

This retrospective study reviewed the medical records of 304 women who were diagnosed as POR and underwent the DuoStim protocol at the Centre for Reproductive Medicine Department of Guangdong Women and Children Hospital for in vitro fertilisation (IVF) and/or intracytoplasmic sperm injection (ICSI) from January 2014 to February 2017. All patients in this study adhered to the Bologna criteria; they each satisfied at least two of the following criteria: advanced maternal age (≥40 years), a history of POR (≤3 oocytes with a conventional stimulation protocol), or abnormal ovarian reserve tests (i.e., antral follicle count < 7 or antimullerian hormone < 1.1 ng/mL) [19]. Exclusion criteria included the presence of known endometrial anomalies, genetic abnormalities, and repeated implantation failure. The study protocol was approved by the institutional ethics committee of Guangdong Women and Children Hospital, and informed was obtained from each patient. This study was conducted in accordance with the principles of the Declaration of Helsinki.

#### IVF treatment and patient groups

**Fig. 1:**
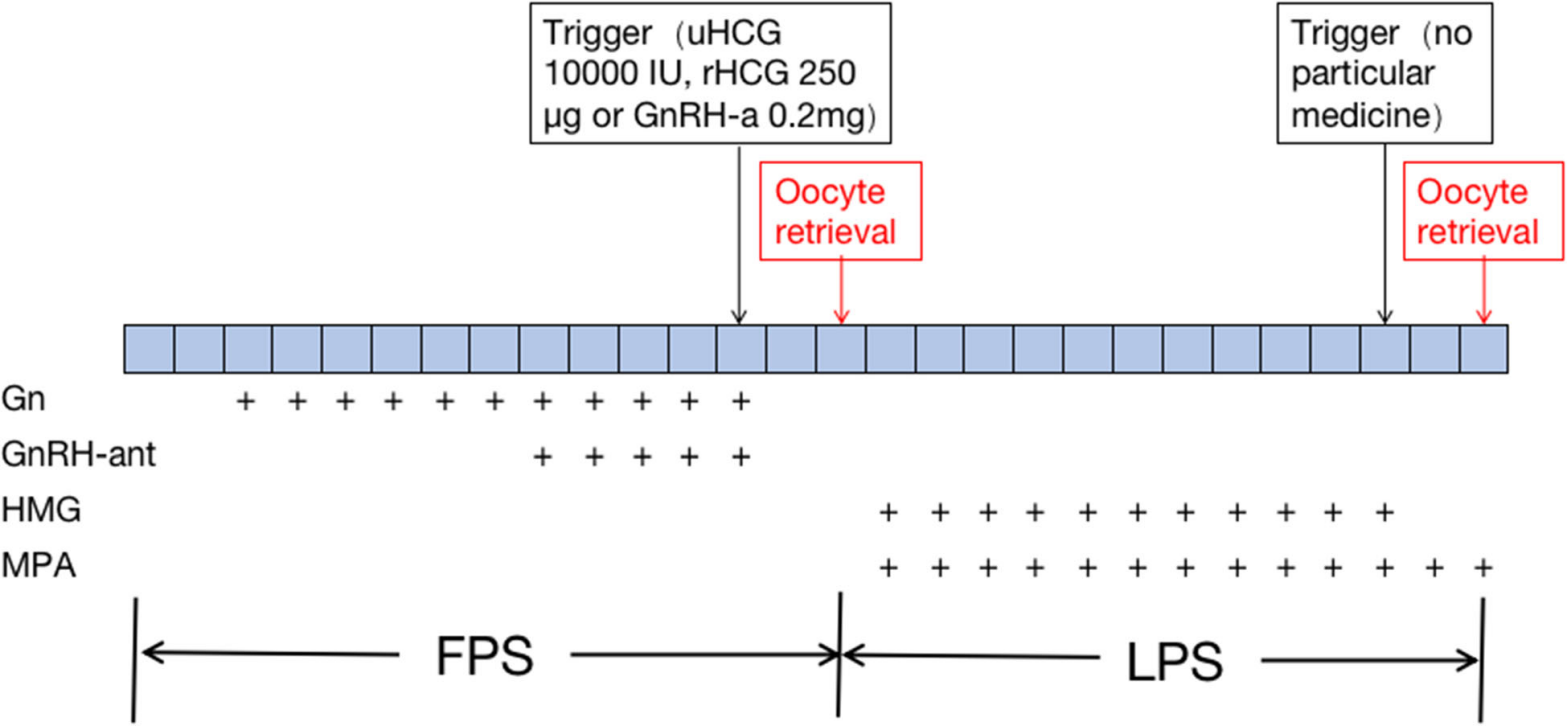
Steps of ovarian stimulation with DuoStim protocol. Gn = Gonadotrophin, GnRH-ant = Gonadotropin-releasing hormone antagonist, HMG = human menopausal gonadotropin, MPA = medroxyprogesterone acetate, FPS = follicular phase stimulation, LPS = luteal phase stimulation, uHCG = urine human chorionic gonadotropin, rHCG = recombinant human chorionic gonadotropin alfafor, GnRH-a = Gonadotropin-releasing hormone agonist

As shown in Fig. 1, FPS was achieved with the administration of a gonadotropin (Gn), including recombinant follicle stimulating hormone (rFSH) or highly purified FSH (HP-FSH) and human menopausal gonadotropin (HMG). The initial dose of Gn ranged from 150 to 300 IU, depending on the basal follicle stimulating hormone (FSH) level, antral follicular count (AFC), and maternal age. The mild ovarian-stimulation regimen, which involves the timely addition of antagonists, was executed during FPS. When 1–3 dominant follicles reached 18–20 mm in diameter, the first trigger medicine was administered. Trigger medicines included urine human chorionic gonadotropin (uHCG; 10,000 IU), recombinant human chorionic gonadotropin (rHCG; 250 μg) and gonadotropin-releasing hormone agonist (GnRH-a; 0.2 mg). Oocytes were retrieved within 34 to 36 h of FPS. One day after the first cycle of oocytes were retrieved, a vaginal ultrasound examination was performed. When the largest follicle diameter was < 10 mm and there were ≥ 2 follicles with diameters between 5 and 10 mm, patients were counselled regarding LPS. LPS was conducted by daily injections of HMG (225 IU). Daily administration of medroxyprogesterone acetate (MPA; 10 mg) was added to avoid oocyte retrieval during menstruation. Again, when 1–3 dominant follicles reached 18–20 mm in diameter, the second trigger medicine of the LPS phase was administered. Oocytes were retrieved within 34 to 36 h of LPS. We compared the data of the FPS phase with the data of the LPS phase in the same patients. Then, all patients were divided into three groups based on the trigger medicine used in FPS or LPS.

### Embryo treatment

Good-quality embryos (Grade A: uniform or slightly uneven with fragmentation of < 10%; Grade B: uniform or non-uniform blastomere size, fragmentation of 10–20%) were frozen on day 3. The embryos that were not of good enough quality for cryopreservation were placed in extended culture until the blastocyst stage. At this stage, on day 5 or day 6, only blastocysts with good morphology were frozen. The embryo quality assessment on day 5 or day 6 was based on the scoring system of Gardner and Schoolcraft, with embryos graded as R3BB considered good blastocysts.

### Frozen–thawed embryo transfer strategy

The frozen-thawed embryo transfer (FET) procedure for cleavage-stage embryos and blastocysts was used. Briefly, embryo vitrification was carried out with the Cyrotop carrier system, with dimethylsulfoxide–ethylene glycol–sucrose as a cryoprotectant. For thawing, embryos were transferred into a series of diluted sucrose solutions (1, 0.5, and 0 mol/L sucrose).

Serum β-HCG levels were measured 12–14 days after the transfer of embryos. Subsequent ultrasound examinations were performed at a gestational age of 7 weeks.

### Outcome measurements

The main outcomes measured were the numbers of dominant follicles, oocytes retrieved, normal fertilised oocytes, cryopreserved embryos, and good quality embryos and blastocysts, as well as the rate of good quality embryos, cryopreserved embryos, and blastocysts, and the implantation and clinical pregnancy rates.

The oocyte yield was defined as the ratio of the total number of collected oocytes to the number of follicles measuring ≥10 mm on the day of oocyte retrieval. The fertilisation rate was defined as the ratio of normal fertilised oocytes (two pronuclei) to the number of oocytes used for fertilisation (for example, the denominator for calculating the fertilisation rate of IVF is all oocytes recovered, while it is the number of MII oocytes for ICSI). The rate of good quality embryos was calculated as the ratio of the number of good quality embryos and the total number of cleaved embryos with two pronuclei, expressed as a percentage. The rate of cryopreserved embryos was calculated as the ratio of the number of available embryos and the total number of cleaved embryos with two pronuclei, expressed as a percentage. The implantation rate was defined as the number of gestational sacs observed divided by the number of embryos transferred. A clinical pregnancy was defined as the identification of a gestational sac 2–3 weeks after the embryo transfer. Early miscarriage was defined as pregnancy ending before 12 weeks of gestation.

The blastocyst formation rate was defined as the number of blastocysts formed divided by the number of embryos cultured. The blastulation rate of embryos of lesser quality was defined as the number of blastocysts formed divided by the number of lesser quality embryos cultured. The pregnancy rate per oocyte retrieval was defined as the pregnancy cycle divided by the number of oocyte retrieval cycles (the number of oocyte retrieval cycles does not include the cycle of mixed embryo transfer or the cycle of unthawed embryos).

### Euploid rate analysis

Finally, among all 304 patients selected for enrolment, only 10 patients underwent the PGT-A cycle. We analysed the embryo euploidy rate of these 10 patients with PGT cycle.

### Statistical analysis

The data were analysed using SPSS version 13 software. Continuous data are presented as an absolute mean with standard deviations (SD). Qualitative data are presented as percentages. The chi-squared test or Fisher’s exact test were used to assess differences between categorical variables and the independent-samples t-test was used to assess differences between continuous variables. Differences in variables between the three trigger medicine groups was analysed using the chi-squared test or Fisher’s exact test and a one-way ANOVA, as appropriate. Statistical significance was set at *p* < 0.05.

## Results

### Baseline cycle characteristics

In this retrospective study, a total of 304 IVF/ICSI cycles were investigated. The clinical features of the patients are shown in Table 1.

**Table 1:** Baseline characteristics of 304 women

Maternal age (years)	Basal FSH (IU/L)	Basal LH (IU/L)	Basal E2 (pg/ml)	Basal AFC	BMI
38.34 ± 4.85	10.17 ± 5.13	4.98 ± 2.31	48.67 ± 41.20	5.19 ± 4.25	22.56 ± 3.15

Values are noted as mean ± SD

*BMI* Body mass index, *E2* estradiol, *AFC* antral follicle count

### Comparison of the FPS and LPS stages

The comparison of the results of the FPS and LPS stages of the DuoStim protocol is shown in Table 2. The total dose of Gn administered during the LPS stage was higher than that in the FPS stage, and the duration of stimulation was longer. Oestrogen (E2), progesterone (P4), and luteinizing hormone (LH) levels measured during the LPS stage on the day that the trigger medicine was administered were higher than those in the FPS stage. The number of follicles at every size on the day that the trigger medication was administered during the LPS stage was significantly higher than that of the FPS stage. The numbers of oocytes retrieved, normal fertilised oocytes, cleaved embryos, cryopreserved embryos, and good quality embryos and the formed blastulation rate of the LPS stage were significantly higher than those of the FPS stage. However, the fertilisation rate, cleaved embryos rate, rate of cryopreserved embryos, and rate of good quality embryos were not significantly different between the two stages of the DuoStim protocol. Of the 304 patients included in this study, 53 patients were transplanted with embryos obtained from the FPS stage, and 110 were transplanted with embryos obtained from the LPS stage. The remaining 141 patients did not undergo embryo transplantation. The final pregnancy rates (including the pregnancy rate per FET and pregnancy rate per oocyte retrieval) and implantation rates obtained using embryos from the LPS stage were higher than those using embryos from the FPS stage. There was no significant difference in the rate of spontaneous abortion.

**Table 2:** Comparison of the FPS and LPS stage within DuoStim protocol

Characteristic	FPS stage *n* = 304	LPS stage *n* = 304	*P* value
Total dose of Gonadotrophin (IU)	1359.51 ± 1135.84	1678.25 ± 882.64*	0.000
Duration of stimulation (days)	6.10 ± 3.48	7.16 ± 3.12*	0.000
E2 on HCG day (pg/ml)	669.27 ± 527.86	1377.95 ± 1155.53*	0.000
P4 on HCG day (ng/ml)	0.63 ± 0.95	14.98 ± 14.35*	0.000
LH on HCG day (IU/L)	5.75 ± 5.50	3.94 ± 4.80*	0.000
Follicles≥14 mm on HCG day	1.46 ± 0.85	3.27 ± 2.94*	0.000
Follicles 10-13 mm on HCG day	0.61 ± 1.01	1.39 ± 2.06*	0.000
Follicles≤10 mm on HCG day	2.07 ± 1.47	4.66 ± 4.49*	0.000
No. of oocyte retrieved	1.71 ± 1.30	3.58 ± 4.54*	0.000
No. of normal fertilized oocyte	1.09 ± 0.91	2.25 ± 2.85*	0.000
No. of cleaved embryos	1.05 ± 0.91	2.20 ± 2.80*	0.000
No. of cryopreserved embryos	0.90 ± 0.78	1.82 ± 2.54*	0.000
No. of good quality embryos	0.79 ± 0.76	1.62 ± 2.40*	0.000
Fertilization rate (%)	62.43%	62.50%	0.978
Cleaved embryos rate (%)	98.77%	98.24%	0.531
Rate of cryopreserved embryos (%)	85.93%	82.63%	0.188
Rate of good quality embryos (%)	74.69%	73.80%	0.766
Formed blastulation rate (%)	48.48%	67.42%*	0.037
Lesser quality embryos formed blastulation rate (%)	23.08%(3/13)	32.08%(17/53)	0.527
Pregnancy rate per FET (%)	26.42%(14/53)	47.27%(52/110)*	0.011
Pregnancy rate per oocyte retrieval (%)	7.37%(14/190)	24.41%(52/213)*	0
Implantation rate (%)	14.48% (21/145)	29.81% (79/265)*	0.001
Spontaneous abortion rate (%)	42.85%(6/14)	21.15%(11/52)	0.099

Data are presented as number (%) unless otherwise indicated. Independent t-test was used to compare continuous variables, and Chi-squared test or Fisher’s exact test for categorical ones

*Compared with FPS Group, *P* < 0.05

### Outcomes of different trigger medicines used during the FPS stage

The patients were divided into three groups based on the first trigger medicine used in FPS: uHCG (*n* = 178), rHCG (*n* = 101), and GnRH-a (*n* = 25) (Table 3). There were no significant differences in baseline characteristics of these groups, however, the total dose of Gn administered was significantly different. Also, there were no significant differences in the numbers of oocyte retrieved, fertilised oocytes, cleaved embryos, cryopreserved embryos, or good quality embryos between the three groups at the FPS stage. The fertilisation rates, cleaved embryos rates, rate of cryopreserved embryos, and good quality embryo rate were also not significantly different between the three groups, nor were the pregnancy rates, implantation rates, or spontaneous abortion rates at the FPS stage.

**Table 3:** Comparison of outcomes at FPS stage of different trigger medicine at FPS stage

Characteristic	uhCG 10,000 IU	rhCG 250 μg	GnRha 0.2 mg	*P* value
Number	178	101	25	
AFC	5.30 ± 4.19	5.57 ± 4.80	3.96 ± 2.52	0.248
FSH (IU/L)	10.22 ± 5.51	9.88 ± 4.71	9.88 ± 3.12	0.841
Matemal age (years)	38.30 ± 5.11	38.0 ± 4.92	38.04 ± 5.67	0.884
Total dose of gonadotrophin (IU)	1524.27 ± 1298.85	1183.04 ± 813.58	906.00 ± 747.78	0.006
Duration of stimulation (days)	6.42 ± 3.70	5.98 ± 3.16	4.24 ± 2.59	0.012
follicle of >10 mm on HCG day	2.06 ± 1.44	2.18 ± 1.64	1.71 ± 0.62	0.367
No. of oocyte retrieved	1.73 ± 1.30	1.72 ± 1.37	1.48 ± 0.96	0.66
No. of normal fertilized oocytes	1.13 ± 0.87	1.03 ± 1.00	1.08 ± 0.78	0.669
No. of cleaved embryos	1.08 ± 0.88	1.01 ± 1.00	1.00 ± 0.82	0.773
No. of cryopreserved embryos	0.93 ± 0.80	0.87 ± 0.77	0.84 ± 0.62	0.745
No. of good embryos	0.80 ± 0.78	0.76 ± 0.75	0.80 ± 0.65	0.929
Fertilization rates (%)	63.64%	58.62%	70.27%	0.327
Cleaved embryos rates (%)	98.47%	100%	96.15%	0.238
Rate of cryopreserved embryos (%)	86.01%	86.27%	84.00%	0.957
Rate of good embryos (%)	73.58%	75.49%	80.00%	0.766
the pregnancy rate per oocyte retrieval (%)	8.03%(9/112)	6.35%(4/63)	6.67%(1/15)	0.914
Pregnancy /FET (%)	29.03%(9 /31)	22.22%(4 /18)	25.00%(1 / 4)	0.871
Implantation rate (%)	16.00%(12/75)	13.79%(8 /58)	8.33%(1 /12)	0.768
Spontaneous abortion rate (%)	44.44%(4 /9)	50.00%(2/4)	0.00%(0/1)	0.656

Data are presented as number (%) unless otherwise indicated. Independent t-test was used to compare continuous variables, and Chi-squared test or Fisher’s exact test for categorical ones

*Compared with uHCG 10,000 IU Group, *P* < 0.05

The outcomes of the LPS stage between the groups that received different trigger medicines during the FPS stage were also compared (Table 4). There were no significant differences in the numbers of oocytes retrieved, fertilised oocytes, cleaved embryos, cryopreserved embryos, or good quality embryos among the three groups. There were also no significant differences in the fertilisation rates or cleaved embryos rates. However, the rates of cryopreserved embryos and good quality embryos obtained in the LPS stage in patients who were first administered rHCG or GnRH-a during the FPS stage were significantly higher than those who were first administered uHCG during the FPS stage. There were no significant differences between patients who received rHCG and those who received GnRH-a during the FPS stage. There were no significant differences in the pregnancy rate, implantation rate, or spontaneous abortion rate among the three groups.

**Table 4:** Comparison of outcomes at LPS stage of different trigger medicine at FPS stage of DuoStim protocol

Characteristic	uHCG 10,000 IU *n* = 178	rHCG 250 μg *n* = 101	GnRH-a 0.2 mg *n* = 25	*P* value
AFC	5.10 ± 4.00	5.33 ± 4.83	4.29 ± 2.51	0.552
FSH (IU/L)	10.33 ± 5.47	9.99 ± 4.78	10.81 ± 4.77	0.759
Matemal age (years)	38.41 ± 5.08	37.98 ± 4.96	38.08 ± 5.64	0.784
Total dose of Gn (IU)	1619.52 ± 813.79	1695.50 ± 820.23	2027.40 ± 1410.64	0.093
Duration of stimulation (days)	6.97 ± 2.89	7.21 ± 2.95	8.32 ± 4.84	0.127
follicle of >10 mm on HCG day	4.65 ± 4.08	4.78 ± 5.40	4.28 ± 3.29	0.884
No. of oocyte retrieved	3.61 ± 3.85	3.64 ± 5.88	3.08 ± 2.41	0.848
No. of fertilized oocytes	2.31 ± 2.26	2.19 ± 3.88	2.12 ± 1.62	0.923
No. of cleaved embryos	2.25 ± 2.21	2.14 ± 3.81	2.08 ± 1.58	0.931
No. of cryopreserved embryos	1.78 ± 1.89	1.90 ± 3.57	1.76 ± 1.30	0.918
No. of good quality embryos	1.56 ± 1.79	1.72 ± 3.36	1.64 ± 1.29	0.865
Fertilization rates (%)	63.45%	59.51%	68.83%	0.227
Cleaved embryos rates (%)	98.04%	98.63%	98.11%	0.864
Rate of cryopreserved embryos (%)	79.00%	88.89%*	84.62%*	0.008
Rate of good quality embryos (%)	69.50%	80.56%*	78.85%*	0.008
Pregnancy rate per oocyte retrieval (%)	26.23%(32/122)	20.00%(14/70)	28.57%(6/21)	0.562
Pregnancy rate per FET (%)	46.38%(32/69)	48.28%(14/29)	50.00%(6/12)	0.966
Implantation rate (%)	27.84%(49/176)	33.90% (20/59)	33.33% (10/30)	0.614
Spontaneous abortion rate (%)	21.88%(7/32)	14.29%(2/14)	33.33%(2/6)	0.625

Data are presented as number (%) unless otherwise indicated. Independent t-test was used to compare continuous variables, and Chi-squared test or Fisher’s exact test for categorical ones

*Compared with uHCG 10,000 IU Group, *P* < 0.05

### Outcomes of different trigger medicines used during the LPS stage

The patients were also divided into three groups based on the first trigger medicine used in LPS: uHCG (*n* = 196), rHCG (*n* = 93), and GnRH-a (*n* = 15) (Table 5). There were no significant differences in baseline characteristics among the three groups. Also, there were no significant differences in the numbers of oocytes retrieved, fertilised oocytes, cleaved embryos, cryopreserved embryos, or good quality embryos among the three groups. There were no significant differences in fertilisation rates or cleaved embryos rates, however, the rates of cryopreserved embryos and good quality embryos in the patients who received rHCG and GnRH-a first during the LPS stage were significantly higher than those in the patients who received uHCG first during the LPS stage. There was no significant difference between patients who received rHCG and patients who received GnRH-a. There was no significant difference between the three groups in the pregnancy rate, implantation rate, or spontaneous abortion rate.

**Table 5:** Comparison of outcomes at LPS stage of different trigger medicine at LPS stage of DuoStim protocol

Characteristic	uhCG	rhCG	GnRha	*P* value
Number	196	93	15	
AFC	4.89 ± 3.89	5.62 ± 4.89	4.87 ± 3.44	0.385
FSH (IU/L)	10.14 ± 4.93	10.18 ± 5.35	12.22 ± 7.06	0.321
Matemal age (years)	38.20 ± 4.83	38.10 ± 5.49	39.67 ± 5.70	0.531
Total dose of gonadotrophin (IU)	1633.29 ± 854.97	1791.68 ± 932.68	1570.00 ± 918.76	0.325
Duration of stimulation (days)	7.04 ± 2.94	7.47 ± 3.37	6.93 ± 3.81	0.527
follicle of >10 mm on HCG day	4.32 ± 4.07	5.38 ± 5.38	4.69 ± 3.17	0.176
No. of oocyte retrieved	3.39 ± 4.32	4.08 ± 5.17	2.93 ± 2.71	0.419
No. of fertilized oocytes	2.16 ± 2.79	2.48 ± 3.14	1.93 ± 1.58	0.614
No. of cleaved embryos	2.11 ± 2.74	2.45 ± 3.08	1.80 ± 1.42	0.531
No. of cryopreserved embryos	1.61 ± 2.38	2.27 ± 2.93	1.67 ± 1.35	0.118
No. of good embryos	1.43 ± 2.31	2.03 ± 2.68	1.67 ± 1.35	0.138
Fertilization rates (%)	63.16%	60.95%	65.91%	0.694
Cleaved embryos rates (%)	98.33%	98.70%	93.10%	0.095
Rate of cryopreserved embryos (%)	76.51%	92.54%*	92.59%	0
Rate of good embryos (%)	67.55%	82.89%*	92.59%*	0
Pregnancy rate per oocyte retrieval (%)	24.63% (33/134)	27.27%(18/66)	7.69%(1/13)	0.322
Pregnancy /FET (%)	47.76% (32/ 67)	54.20%(19/ 35)	12.5%(1 /8)	0.101
Implantation rate (%)	28.25% (50/177)	37.33%(28/75)	7.69%(1 / 13)	0.072
Spontaneous abortion rate (%)	18.75% (6 / 32)	21.05%(4/19)	100.00%(1/ 1)	0.147

Data are presented as number (%) unless otherwise indicated. Independent t-test was used to compare continuous variables, and Chi-squared test or Fisher’s exact test for categorical ones

*Compared with uHCG 10,000 IU Group, *P* < 0.05

### Euploid rate analysis

Among the 304 POR patients, 10 patients underwent a PGT-A cycle, including 5 patients younger than 35 years old and 5 patients older than or equal to 35 years old. A total of 20 embryos were obtained from 10 patients, of which 8 embryos were from the FPS stage and 12 embryos were from the LPS stage. The proportions of aneuploidy embryos in the FPS group and the LPS group were 75.00% (6/8) and 33.33% (4/12), respectively, and there was no statistical difference. The ratios of transferable embryos in the FPS group and LPS group were 25.00% (2/8) and 58.33% (7/12), respectively, and there was no statistical difference (Table 6).

**Table 6:** Euploid rate analysis

Characteristic	FPS group	LPS group	t/c^2^ value	*P* value
number	4	6		
Proportion of patients≥35 years old	50%(2/4)	50%(3/6)	0	1
Aneuplidy embryo rate	75.00%(6/8)	33.33%(4/12)	3.333	0.068
Chromosome mosaicism embryo rate	0.00%(0/8)	8.33%(1/12)	0.702	0.402
Transferable embryo rate	25.00%(2/8)	58.33%(7/12)	2.155	0.142

Data are presented as number (%). Chi-squared test or Fisher’s exact test was used for categorical variables

## Discussion

We found that the numbers of oocytes retrieved, normal fertilised oocytes, cleaved embryos, cryopreserved embryos, and good quality embryos during the LPS stage are significantly higher than those during the FPS stage in POR patients undergoing the DuoStim protocol. We also found that the trigger medicines used during the FPS stage did not affect the outcomes of that stage. The rates of cryopreserved embryos and good quality embryos resulting from the use of rHCG and GnRH-a as trigger medicines were significantly higher than those obtained when uHCG was used as a trigger medicine.

Overall, the number of oocytes obtained and the rate of good quality embryos were higher in the LPS stage than in the FPS stage. This is consistent with previous studies [10–16]. Embryos obtained at the LPS stage may result in more favourable outcomes due to several factors. The high oestrogen and progesterone levels of the LPS stage may result in more synchronous follicular development and promote the proliferation of FSH receptors in granular cells [20]. Animal studies have reported that high oestrogen and progesterone levels at the LPS stage may lead to an increase in angiogenic factors, thereby promoting the sensitivity of granulocytes to FSH [22]. Another study found no significant differences in the miRNA of the follicular fluid in patients at the LPS and FPS stages, however, this study requires verification due to the limitations of the miRNA detection screening index and its limited sample size [23]. No differences have been reported in the final neonatal birth defect rate in embryos obtained during the FPS or LPS stage [24]. Taken together, the results of these studies suggest that obtaining oocytes at the LPS stage results in a higher number of embryos, however, future studies are required to confirm this conclusion.

When GnRH-a was administered as a trigger medicine, the rate of good quality embryos was higher, regardless of whether it was administered during the FPS or LPS stage. This may be explained by the fact that GnRH-a has a smaller flare up effect, which can improve the synchronisation of follicular development [25]. The flare up effect of GnRH-a will results in a slight increase in endogenous FSH levels, which may promote the growth of follicles with a diameter of 4 mm or less. At the same time, the elevated FSH concentration is lower than the threshold that allows periodic follicle recruitment, rendering follicles with a diameter of 7.5–10 mm unable to begin to develop. GnRH-a may also regulate the cascade of follicular development events before follicles mature [26, 27]. The local regulation factors influenced by GnRH-a include anti-Mullerian hormone (AMH) and inhibin B, which are secreted by granulocytes. AMH inhibits the initiation of primitive follicular growth in the ovarian reserve. In contrast to AMH, all follicles produce inhibin B, which helps reduce FSH prior to follicular maturity [28]. However, these hypotheses have yet to be confirmed, and the role of endocrine and paracrine factors in the mechanism of follicle recruitment that regulates the growth of the anovulatory tide in the ovarian cycle requires further investigation.

The advantages of rHCG as a trigger drug observed in this study were not anticipated by our team. rHCG is an HCG preparation extracted from Chinese hamster ovary cells using recombinant technology [29, 30]. Compared with uHCG extracted from pregnant women’s urine, rHCG is more pure, has a higher batch-to-batch consistency, and can be injected subcutaneously [31, 32]. In addition, because rHCG is not available in all countries, its pharmacology, pharmacokinetics, and the differences after application to patients of different races are unknown. A Japanese study showed that the potential differences in the pharmacokinetic profiles of rHCG between Japanese and Caucasian patients cannot be ignored [33].

It has been reported that the number of oocytes retrieved per patient and the clinical pregnancy rates resulting from the use of the GnRH-a long protocol, as in COS, were similar when rHCG and uHCG were used [34, 35]. rHCG has been reported to have lower incidences of injection site reactions (such as redness and swelling) than uHCG, [36] which may be attributed to fewer contaminating proteins in rHCG. In addition, a reduction in patient complaints of redness and swelling were observed after uHCG was switched to rHCG [37]. Therefore, the use of rHCG or GnRH-a as a trigger medicine in the FPS stage of the DuoStim protocol maybe better than the use of uHCG. However, more clinical and pharmacological research will be needed to determine more benefits of the use of rHCG and GnRH-a in the DuoStim protocol.

Our study has a unique design. First, we used an antagonist regimen in the follicular phase and a progesterone-primed ovarian stimulation (PPOS) regimen in the luteal phase to prevent the surge of the LH peak. There has been controversy about whether the use of MPA in the PPOS program will affect the outcome of pregnancy. However, the literature in recent years has all related to the safety and effectiveness of the PPOS scheme [38]. It is suggested that the numbers of blastocysts and euploid blastocysts per patient and the number of euploid embryos per injected oocyte are similar for patients undergoing the PPOS program and for those undergoing COS with GnRH antagonists [39]. Flexible progestin-primed ovarian stimulation (FPPOS) with MPA seems to be an effective choice for preventing premature ovulation in women undergoing ovarian stimulation without compromising oocyte quality [40]. Kuang et al. demonstrated that PPOS had a more robust control for preventing the premature rise of LH than GnRH antagonists in poor responders. Moreover, the comparison of the data in our article suggests that the ratio of available embryos and quality embryos for FPS and LPS is not statistically significant.

We used different types of Gn in the follicular and luteal phases. As the DuoStim scheme for COS has relatively little literature support, the Gn selected by different studies, especially the Gn in the luteal phase, are not consistent [12, 41]. We have not found evidence in the literature to support the use of different types of Gn in different follicular waves. The use of different types of Gn in different follicular waves requires further investigation. The DuoStim program has a high cycle cancellation rate during the implementation process, which may be due to the patient’s own personal reasons or the lack of a second follicular wave development in the luteal phase. Therefore, we used HMG in the luteal phase as it is more economical than rFSH. In addition, studies suggest that although rFSH may be administered at a lower dosage over fewer days during ovulation induction, the resulting pregnancy rate is not better than that of HMG [20, 42]. We believe that it is safe and economical to use HMG as a Gn drug during the luteal phase.

We also compared the aneuploidy rate of embryos in POR patients after DuoStim protocol, although the number of cases was limited. As the maternal age increases, the probability of abnormal meiosis during oogenesis or abnormal mitosis after fertilization is significantly increased, and the chance of complete chromosome deletion or addition in the embryo is increased [43, 44]. The incidence of chromosome abnormalities increases from 25% in women around the age of 20 to 65% in women over the age of 40 [45, 46]. However, whether maternal age is related to the risk of fragmented sex chromosome deletion or addition in embryos is still inconclusive, and requires confirmation by a large study. One study analysed the biopsy results of 15,169 blastocysts from women of different ages, and found that the incidence of aneuploidy in women aged 26 to 30 was the lowest, and the proportion of aneuploidy embryos increased significantly after the age of 35 [46]. Aneuploidy is an important factor in the failure of assisted pregnancy and spontaneous abortion in women with advanced maternal age. However, despite the limited sample size in this study, we found that the developmental potential of LPS and FPS embryos are similar. LPS embryos are not affected by high progesterone levels. This is consistent with the results of previous studies [2, 20].

The ESHRE (the european society of human reproduction and embryology) states that the DuoStim protocol should be used only for research.. However, we believe that DuoStim can be safely and effectively used in patients who are POR. Their suggestion may be based on the safety of the DuoStim protocol. Baerwald et al. has proposed three theories of follicle recruitment [28]_:_ the theory of continuous recruitment, single recruitment theory, and the wave theory. The theory of continuous recruitment includes the interval between two ovulations [47]. During this time, the follicles begin to grow continuously and gradually disappear, and the dominant follicle is randomly selected after the corpus luteum has degenerated. The single recruitment theory involves a single group of follicles that start to grow after the corpus luteum degenerates, among which the dominant follicle is selected. The wave theory includes at least two groups of sinus eggs that are recruited. In each ovarian cycle, the dominant follicle originates only from the main (or ovulation) wave. There is no correlation between the average capacity of each patient’s MII oocytes recruited from the follicular phase and the luteal phase of the same follicular cycle, indicating that the two cohorts are independent. In addition, euploid blastocysts may be produced at any of the two stages of the ovarian cycle, which suggests that non-dominant follicles may be capable and will develop in a random manner. In other words, the advantages of the follicle may not automatically reflect its function. Therefore, we believe that the DuoStim protocol has advantages in POR patients, and there are no reports of safety concerns. The DuoStim protocol can be used as a method for COS in POR patients, however, further research is required to determine the feasibility of this expensive protocol.

The adverse reactions and complications of the accumulation of a high number of oocytes in a relatively narrow cycle time during the DuoStim protocol [48], and the short-term and long-term effects on the patient and the embryos require additional research. The length of time between the two stimulation schemes is very short, and the biological markers and microenvironment stability of the twice-obtained oocytes remain to be studied. The physiological aspects of the follicle recruitment theory also require further research. A comparison of the transcription and microenvironment aspects of the follicular fluid of the FPS and LPS stages, and an evaluation of the follicular fluid of embryos obtained at the LPS stage after different trigger schemes should be performed. Single and double trigger schemes should also be compared.

This is the first study to investigate the effects of the trigger medicine on oocyte retrieval in the DuoStim protocol. However, our study is not without limitations. This is a retrospective study and the sample size is limited. Comparing our results to those of previous studies is challenging due to the diversity of COS protocols.

## Conclusions

Obtaining more and higher-quality oocytes and embryos in a short period of time while shortening the pregnancy time is crucial for POR patients. The high oocyte retrieval of the DuoStim protocol is suitable for patients who need to accumulate embryos, such as patients with cancer. The administration of GnRH-a or rHCG as the trigger medicine may be better than uHCG during both the FPS and LPS stages for POR undergoing the DuoStim protocol. We found that the number of oocytes obtained in the LPS stage was greater than that in the FPS stage.

## Data Availability

The datasets used and analysed during the current study are available from the corresponding author on reasonable request.

## Abbreviations

POR: Poor ovarian responder
ART: Assisted reproduction technology
COS: Controlled ovarian stimulation
MII: Metaphase II
FPS: Follicular phase stimulation
LPS: Luteal phase stimulation
PGT-A: Preimplantation genetic testing for aneuploidy
Gn: Gonadotropin
IVF: In vitro fertilisation
ICSI: Intracytoplasmic sperm injection
rFSH: Recombinant follicle stimulating hormone
HP-FSH: Highly purified FSH
HMG: Human menopausal gonadotropin
FSH: Follicle stimulating hormone
AFC: Antral follicular count
uHCG: Urine human chorionic gonadotropin
rHCG: Recombinant human chorionic gonadotropin
GnRH-a: Gonadotropin-releasing hormone agonist
MPA: Medroxyprogesterone acetate
E2: Oestrogen
P4: Progesterone
LH: Luteinizing hormone
AMH: Anti-Mullerian hormone
PPOS: Progesterone-primed ovarian stimulation
FPPOS: Flexible progestin-primed ovarian stimulation
ESHRE: The european society of human reproduction and embryology
